# Uncommon Twist: Femoral Neck Stress Fracture in a High-Performance Athlete With an Incidental Diagnosis of Femoroacetabular Impingement

**DOI:** 10.7759/cureus.59224

**Published:** 2024-04-28

**Authors:** Ana L Melero-Pardo, Tatiana C Pimentel-Soler, Carlos R Benitez-Colón

**Affiliations:** 1 Medicine, Universidad Central del Caribe, San Juan, PRI

**Keywords:** femoroacetabular impingement, sports medicine, orthopedics, internal fixation, femoral neck stress fractures

## Abstract

Femoral neck stress fractures (FNSFs) are rare but significant injuries, often leading to delayed diagnosis due to nonspecific symptomatology. This case report presents a 30-year-old professional surfer who experienced acute groin pain during a half marathon, ultimately diagnosed with a left intertrochanteric line femur stress fracture and with femoroacetabular impingement (FAI) in the opposite hip. Despite being physically fit, his presentation challenges the prevailing notion that FNSFs predominantly occur in military personnel or the elderly. The patient underwent surgical left hip osteosynthesis without complications. This case highlights the importance of early suspicion of FNSFs in young, active individuals and emphasizes the need for comprehensive evaluation to prevent complications like osteonecrosis and malunion. It underscores the value of a broad differential diagnosis and timely intervention in optimizing outcomes, especially in the context of rising high-impact sports participation.

## Introduction

Femoral neck stress fractures (FNSFs) are rare injuries that often result in delayed diagnosis due to initial symptoms being overlooked [1]. They represent approximately 1%-2% of all femoral neck fractures, 3%-5% of all stress fractures, and account for about 3% of sport-related events [2,3]. FNSFs demonstrate a bimodal distribution, affecting both a younger, active demographic and an older population with potentially compromised bone quality or impaired bone repair mechanisms [4]. In the younger cohort, FNSFs are mainly observed among military personnel and high-performing athletes, commonly linked with a rapid increase in physical activity and endurance exercise [1,2,5-7].

Several studies have pinpointed the primary risk factors for FNSFs, including female gender with a peak age range over 60, poor baseline physical fitness, and decreased bone mineral density [3,5]. Despite their clinical significance, FNSFs often go undiagnosed until advanced stages due to delayed symptom recognition [8]. Patients typically present with a gradual onset of aching groin pain that worsens with high-impact or weight-bearing exercise and improves with rest. However, diagnostic differentials such as muscle strains, hip osteoarthritis, avascular necrosis of the femoral head, synovitis, and labral tears can make it challenging to promptly diagnose femoral neck stress fractures. Over the years, various classification systems for FNSFs have been developed, with the Garden and Fullerton and Snowdy classifications being the most widely accepted [9,10]. The Garden classification divides fractures into four types based on displacement, while the Fullerton and Snowdy classification categorizes them based on displacement, angulation, and rotation [9,10]. These classifications are crucial for guiding treatment strategies and predict prognosis based on the type and severity of the fracture.

Treatment of FNSFs depends on the fracture morphology identified by magnetic resonance imaging (MRI) and the presence of an intra-articular effusion [11]. Non-surgical management involves a period of non-weight bearing followed by a gradual return to activity, while surgical management includes fracture fixation to prevent further progression. Failure to promptly diagnose and treat FNSFs can lead to complications such as osteonecrosis of the femoral head and malunion [11]. These complications may necessitate further surgical interventions, prolonged rehabilitation, and may have long-term implications on the patient's overall health and well-being. This case report highlights the importance of considering FNSFs in the differential diagnosis of acute groin or hip pain in a 30-year-old, physically active individual.

## Case presentation

**Figure 1:**
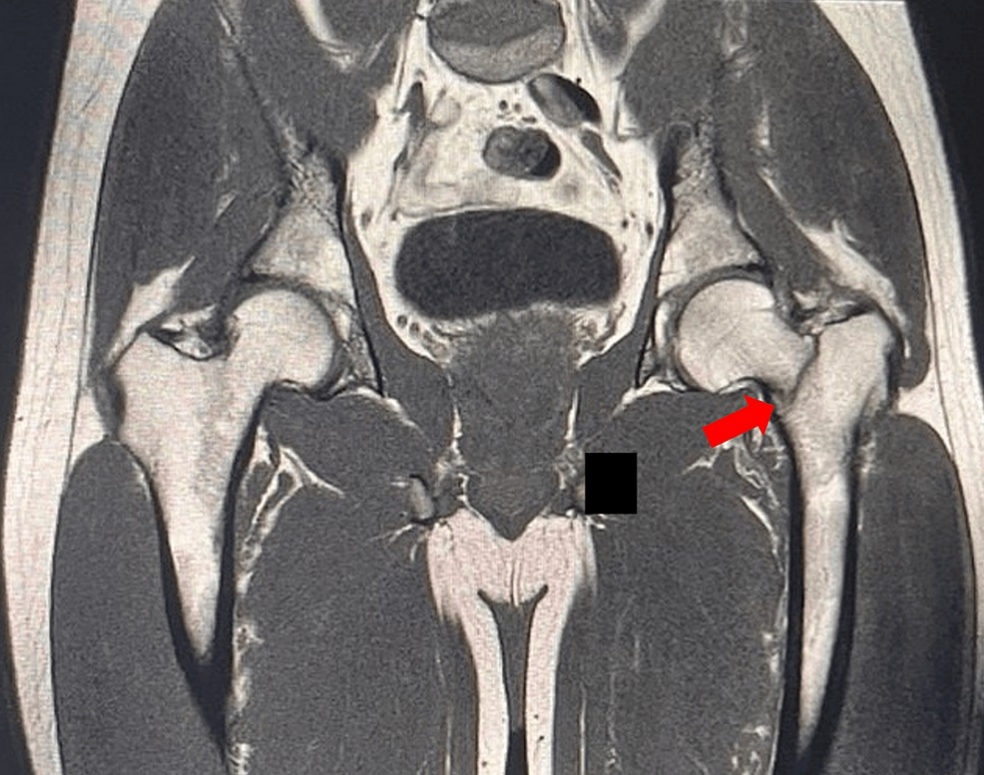
MRI of a Stress Fracture in the Left Femoral Neck MRI image showing a stress fracture of the femoral neck in the left leg, highlighted by a red arrow. A black box is placed over the radiologist's cursor to prevent distraction.

**Figure 2:**
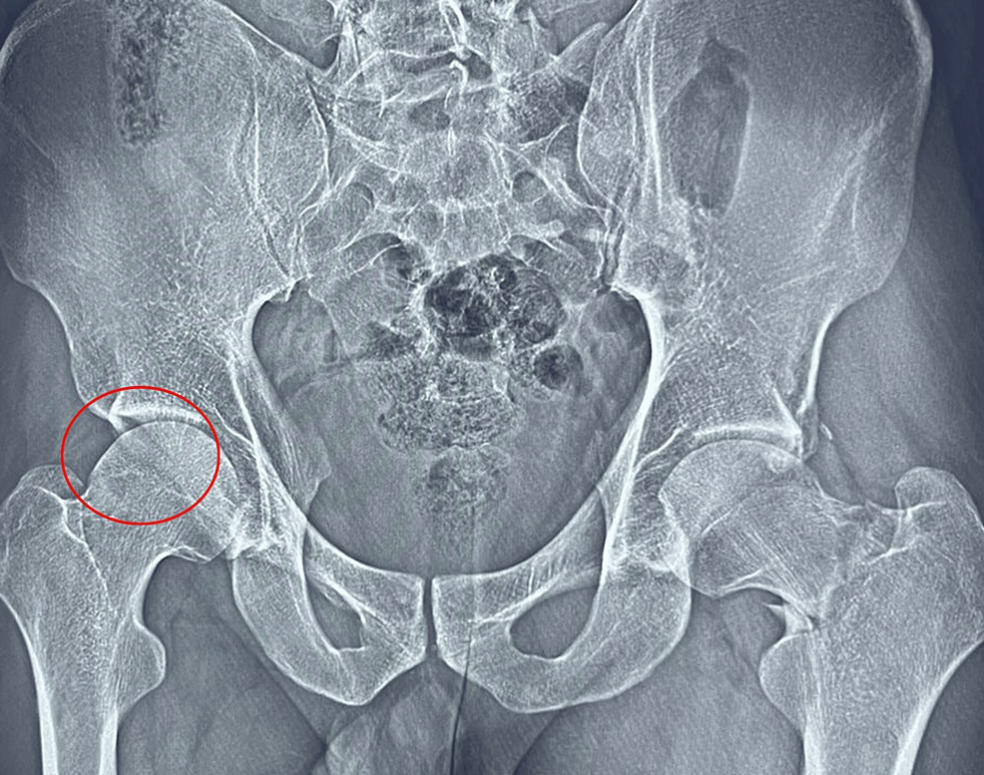
Anteroposterior Pelvic Radiograph Demonstrating Femoroacetabular Impingement (FAI) in the Right Hip Anteroposterior pelvic radiograph illustrating femoroacetabular impingement (FAI) in the right hip. The red circle outlines the overhanging acetabular rim, a characteristic feature of pincer impingement.

**Figure 3:**
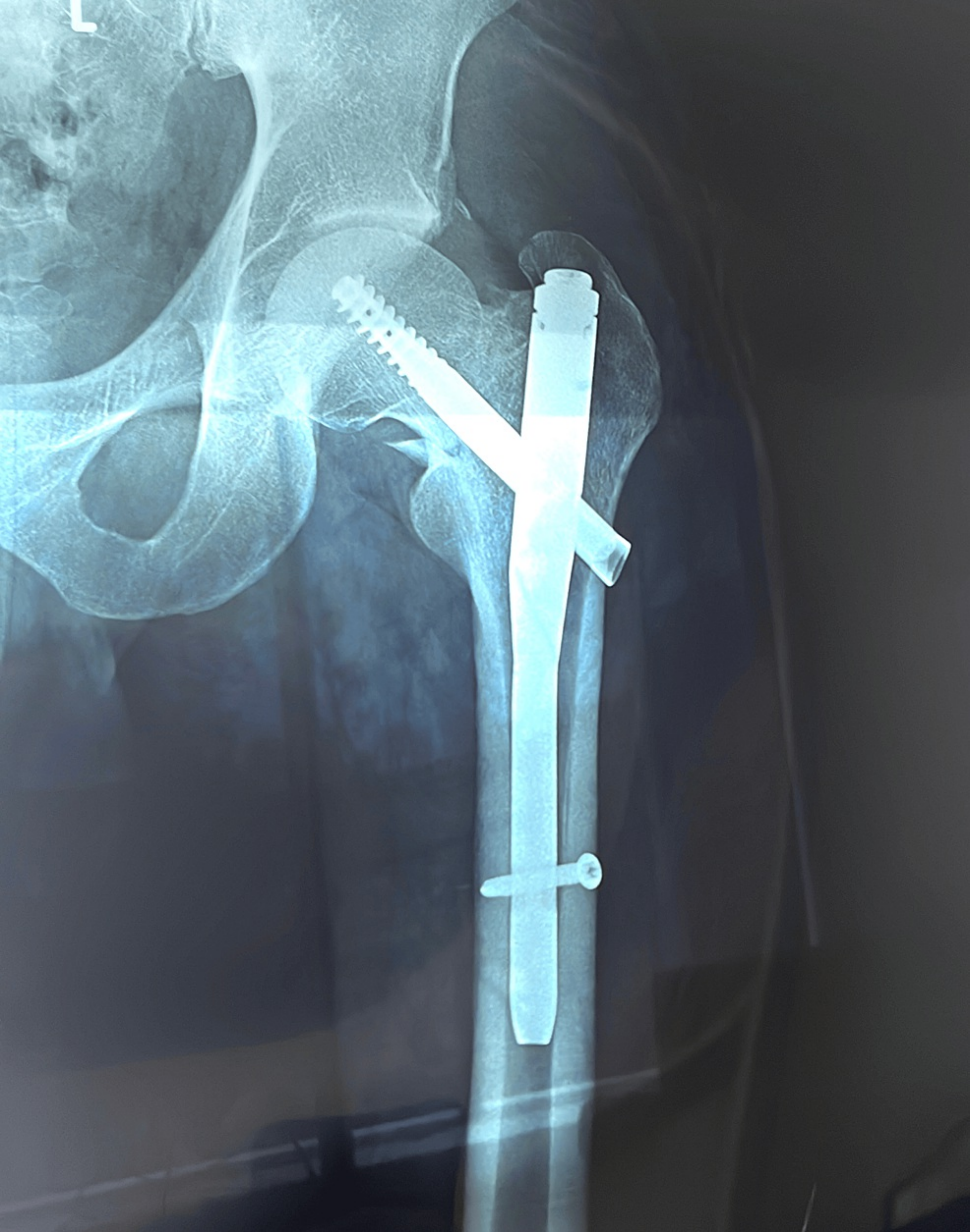
Anteroposterior Radiograph of the Left Femur Post-Internal Fixation with Osteosynthesis

A 30-year-old, 160-pound, otherwise-healthy, male professional surfer who has been actively surfing since the age of three and surfs with a goofy-foot stance (right foot forward) presented with acute onset of symptoms during a half marathon. He described experiencing a snapping sensation in the left inguinal region, followed by severe pain upon ambulation, rated 7/10 on the pain intensity scale. The pain was described as incapacitating, exacerbated by movement, and radiating to the left gluteal region. Due to the severity of his symptoms, he sought urgent medical evaluation. His medical history includes arthroscopic surgery for a right medial meniscal tear 11 years ago. There is no family history of femoral neck fractures or similar events, and the patient is not currently taking any medications. A comprehensive evaluation, including physical examination and pelvic MRI, was conducted. The MRI revealed findings consistent with a stress fracture involving the left intertrochanteric line of the femur, accompanied by joint effusion (Figure 1). The patient was incidentally diagnosed with femoroacetabular impingement (FAI) of the right hip based on imaging findings (Figure 2). The patient subsequently underwent a surgical procedure involving left hip osteosynthesis with internal fixation, which was performed without intraoperative complications (Figure 3). Postoperatively, the patient's recovery was satisfactory.

## Discussion

This case report underscores several unique aspects that contribute to its clinical significance. Literature reports that running sports are considered the leading cause of stress fractures of the lower extremities in athletes, with a high incidence of these injuries among recreational athletes and in individuals starting physical activity following a sedentary lifestyle [12,13]. In our case, the patient, a young, healthy male professional surfer, presented with an acute onset of severe symptoms during a half marathon, highlighting the potential for FNSFs to occur even in physically fit individuals. Furthermore, this case challenges the common misconception that such fractures are primarily seen in military personnel and the elderly, emphasizing the need for a broader clinical suspicion in the evaluation of acute groin or hip pain in young adults [14,15].

Secondly, the nonspecific nature of symptoms associated with FNSFs, such as gradual onset, atraumatic hip and groin pain exacerbated by exercise and relieved with rest, often leads to delayed diagnosis [11]. In this case, the patient reported a snapping sensation followed by severe pain, which could easily be misattributed to muscle strain or other less severe musculoskeletal injuries. This highlights the importance of maintaining a high index of suspicion for FNSFs and considering them in the differential diagnosis, especially in patients engaged in high-impact physical activities.

The timely recognition and accurate diagnosis of FNSFs are crucial for effective management and prevention of potential complications. Delayed or missed diagnosis can lead to the progression of the fracture, resulting in complications such as osteonecrosis of the femoral head, malunion, and long-term disability [11]. These complications can significantly impact the patient's quality of life, require more invasive treatment strategies, and potentially lead to long-term disability.

Treatment strategies for FNSFs are tailored based on the fracture type and severity. While conservative management, including rest, weight-bearing restriction, and gradual reintroduction of impact exercise, is often sufficient for low-risk fractures, high-risk fractures may necessitate more aggressive approaches [16]. For high-risk femoral neck fractures, more aggressive treatment approaches may include surgical fixation, hip arthroplasty, open reduction and internal fixation (ORIF), dynamic hip screw fixation, and intramedullary nailing [17-20]. In this case, the patient's presentation and imaging findings warranted surgical intervention, specifically left hip osteosynthesis, which was performed without intraoperative complications and resulted in a satisfactory postoperative outcome.

Moreover, the patient was diagnosed with FAI in his right hip, a hip condition characterized by abnormal contact between the femoral head and the acetabulum, leading to potential damage to the hip joint structures [21]. Patients with FAI often present with hip pain, stiffness, and limited range of motion, especially during activities that involve flexing or rotating the hip. The onset of FAI symptoms in athletes is variable, with damage resulting from the cumulative effect of cyclical abnormal wear associated with altered joint morphology [22-24].

Diagnosis of FAI typically involves a combination of clinical evaluation, including a thorough medical history and physical examination, and imaging studies such as X-rays, MRI, and occasionally fluoroscopically guided hip injections to confirm the presence of FAI and determine the type and severity of impingement [25]. While most literature on FAI focuses on athletes in general, particularly those involved in sports that require repetitive hip movements or those at risk of hip injuries due to the nature of the sport, there is limited specific research on surfers with FAI.

Surfing involves dynamic movements and places stress on the hip joint, making it plausible to hypothesize that the repetitive motions and strains associated with this sport could contribute to the development or exacerbation of FAI. Additionally, there is emerging evidence suggesting a link between FAI and femoral neck stress fractures (FNSFs) [26]. Research findings show that patients in the general population with FNSFs have a greater prevalence of bone irregularities linked to FAI [27,28]. However, more research is warranted to establish a definitive association between FAI and FNSFs and understanding the underlying mechanisms.

Therefore, this case report emphasizes the importance of maintaining a high index of suspicion for femoral neck stress fractures, even in physically fit individuals engaged in high-impact activities such as surfing. It underscores the need for a comprehensive approach to patient evaluation, timely diagnosis, and appropriate management to optimize outcomes, prevent complications, and challenge prevailing misconceptions in clinical practice.

## Conclusions

This case report underscores the critical role that clinical suspicion and a comprehensive approach to patient evaluation play in medicine, particularly when confronting atypical presentations of common conditions. The unexpected discovery of femoroacetabular impingement in the opposing leg of a healthy, athletic patient with a femoral neck stress fracture highlights the complexities of human physiology and pathology, emphasizing the need for clinicians to maintain an open-mind approach and consider a broad range of differential diagnoses. As the popularity of running and other high-impact sports continues to rise, this case emphasizes the importance of maintaining a high index of suspicion for femoral neck stress fractures (FNSFs) in young, physically active individuals presenting with acute groin or hip pain. Vigilance in considering FNSFs as a potential diagnosis is crucial for early identification, appropriate management, and optimizing outcomes, facilitating timely return to physical activity, and preventing long-term complications.
